# Repurposing Seleno-L-Methionine as a Pleiotropic Immunomodulatory Agent to Overcome TGF-β1/HIF-Driven Immune Evasion in Clear Cell Renal Cell Carcinoma: Mechanistic Insights and Translational Therapeutic Opportunities

**DOI:** 10.3390/ijms27177858

**Published:** 2026-09-02

**Authors:** Youcef M. Rustum

**Affiliations:** Roswell Park Comprehensive Cancer Center, 666 ELM Street, Buffalo, NY 14202, USA; youcef.rustum@gmail.com

**Keywords:** TGF-β1, HIF1α, HIF2α, SLM, immune evasion, metabolic reprograming, therapeutic potential, ccRCC

## Abstract

Clear cell renal cell carcinoma (ccRCC) is a highly immune-evasive malignancy that exhibits limited and often transient responses to immune checkpoint inhibitors (ICIs) and remains a major challenge for emerging cellular immunotherapies, including chimeric antigen receptor (CAR)-T cells. A defining feature of ccRCC, largely driven by von Hippel–Lindau (VHL) deficiency, is persistent activation of the transforming growth factor-β1 (TGF-β1) and hypoxia-inducible factor (HIF) signaling network. Acting as a central immunometabolic regulatory axis, TGF-β1/HIF promotes angiogenesis, metabolic reprogramming, epigenetic dysregulation, and immune escape through coordinated induction of immunosuppressive mediators, including PD-L1, VEGF, and CTLA-4, resulting in impaired T-cell infiltration, functional exhaustion, and resistance to immunotherapy. Preclinical studies have demonstrated that pharmacologic-dose Seleno-L-methionine (SLM) and its active metabolite, methylseleninic acid (MSA), suppress TGF-β1 and both HIF-1α and HIF-2α, leading to downregulation of multiple downstream immunosuppressive pathways at pharmacologically achievable, non-toxic concentrations. In addition to enhancing the antitumor activity of chemotherapy and VEGF-targeted agents, accumulating evidence suggests that SLM exerts broad immunologic, metabolic, and epigenetic effects that may overcome key mechanisms of therapeutic resistance. This review synthesizes current mechanistic and translational evidence supporting the repurposing of SLM as a first-in-class pleiotropic immunomodulatory agent. Using ccRCC as a model of TGF-β1/HIF-driven immune resistance, we discuss how simultaneous targeting of this central regulatory axis may restore immune surveillance, improve T-cell fitness and trafficking, enhance responses to ICIs and CAR-T cell therapy, and provide a mechanistically rational strategy for the treatment of advanced solid tumors.

## 1. Scope of the Review

This review examines the emerging role of pharmacologic SLM as a pleiotropic immunomodulatory agent that simultaneously targets the TGF-β1/HIF-1α/HIF-2α signaling network, a central driver of immune evasion, metabolic reprogramming, abnormal angiogenesis, and therapeutic resistance in clear cell renal cell carcinoma (ccRCC). Unlike current therapeutic strategies that selectively inhibit individual components of these pathways, SLM suppresses multiple interconnected molecular and cellular processes that collectively sustain the immunosuppressive tumor microenvironment. Importantly, the pharmacologically active doses of SLM evaluated in translational and clinical studies are approximately 20- to 40-fold higher than those used in nutritional supplementation or cancer prevention trials yet have demonstrated a favorable safety profile without significant toxicity to normal tissues. At these doses, SLM functions not as a conventional cytotoxic agent but as a selective regulator of the TGF-β1/HIF signaling axis, resulting in coordinated molecular, epigenetic, metabolic, vascular, and immune reprogramming. We summarize current evidence demonstrating that inhibition of the TGF-β1/HIF axis by SLM downregulates key mediators of immune escape and treatment resistance, including immune checkpoint molecules, angiogenic factors, hypoxia-responsive genes, and epigenetic regulators, while simultaneously promoting vascular normalization, restoration of antitumor immunity, and enhanced therapeutic responsiveness. We also discuss the mechanistic basis by which SLM may improve the efficacy of immune checkpoint inhibitors, anti-angiogenic therapy, and emerging adoptive cellular therapies, including CAR-T cells. Although many of the underlying mechanisms are relevant across solid malignancies, this review focuses on clear cell renal cell carcinoma, whose unique molecular, metabolic, histologic, and immunologic characteristics—including constitutive HIF activation following VHL loss and frequent overexpression of TGF-β1—provide an ideal translational model for investigating TGF-β1/HIF-targeted therapeutic strategies. We propose that ccRCC represents a proof-of-concept disease in which pharmacologic SLM may serve as a novel host-directed therapeutic platform capable of overcoming multiple resistance mechanisms through simultaneous modulation of interconnected oncogenic pathways rather than single-target inhibition.

## 2. Introduction

Clear cell renal cell carcinoma, which accounts for approximately 75–80% of all kidney cancers, is the most common and aggressive histologic subtype. Despite major advances with immune checkpoint inhibitors (ICIs) and vascular endothelial growth factor (VEGF)-targeted tyrosine kinase inhibitors (TKIs), durable responses remain limited, and the 5-year survival rate for metastatic disease remains below 15–20% [1,2,3]. Although ccRCC is characterized by abundant immune-cell infiltration, its TME is paradoxically highly immunogenic yet profoundly immunosuppressive, owing to persistent hypoxia, aberrant angiogenesis, metabolic reprogramming, and dysfunctional antitumor immunity. A defining molecular feature of advanced ccRCC is constitutive activation of HIF-1α and HIF-2α together with overexpression of TGF-β1. These pathways cooperate to promote tumor progression, angiogenesis, metastatic dissemination, immune evasion, and therapeutic resistance. Increasing evidence indicates that reciprocal crosstalk between TGF-β1 and HIFs establishes a self-sustaining metabolic–epigenetic lock, which maintains the malignant phenotype while limiting responsiveness to current therapies.

The TGF-β1/HIF axis orchestrates extensive metabolic remodeling by suppressing key tricarboxylic acid (TCA) cycle enzymes, including succinate dehydrogenase (SDH) and fumarate hydratase (FH). The resulting accumulation of succinate and fumarate inhibits prolyl hydroxylase domain protein-2 (PHD2), thereby stabilizing HIFs, while simultaneously inhibiting ten-eleven translocation (TET) DNA demethylases, leading to persistent epigenetic repression [4,5,6,7,8,9,10]. In parallel, TGF-β1 stabilizes DNA methyltransferase-1 (DNMT1), promoting DNA hypermethylation and silencing genes that regulate mitochondrial metabolism, including carnitine palmitoyl transferase-1A (CPT1A), the rate-limiting enzyme in fatty acid β-oxidation, and additional metabolic regulators involved in lipid homeostasis [11,12,13,14,15,16]. Collectively, these alterations drive lipid accumulation, generate the characteristic clear-cell phenotype, impair mitochondrial oxidative metabolism, and reinforce an immunosuppressive TME. Because currently approved TKIs and ICIs primarily target downstream angiogenic or immune pathways, they do not directly reverse this integrated metabolic–epigenetic program. Consequently, the underlying mechanisms that sustain hypoxia, immune dysfunction, and metabolic plasticity persist, contributing to intrinsic and acquired therapeutic resistance. These observations highlight the need for therapeutic strategies capable of simultaneously restoring metabolic homeostasis and reversing immune suppression.

This review examines the molecular mechanisms by which the TGF-β1/HIF signaling network establishes and maintains the metabolic–epigenetic lock in ccRCC and evaluates emerging evidence supporting its therapeutic disruption. We focus on SLM as a pleiotropic immunometabolic modulator capable of simultaneously suppressing TGF-β1 and HIF signaling, restoring mitochondrial and epigenetic function, normalizing the tumor microenvironment, and enhancing antitumor immunity. Unlike selective pathway inhibitors, pharmacologic SLM acts through multiple complementary mechanisms independent of VHL status and is active in tumors with or without sarcomatoid differentiation. By dismantling the TGF-β1/HIF-driven metabolic barrier, SLM has the potential to sensitize ccRCC to VEGF-targeted TKIs, immune checkpoint blockade, and emerging CD70- and Fibroblast growth factor-inducible 14 (Fn14)-directed Chimeric Antigen Receptor (CAR)-T cell therapies.

Although clinical studies combining SLM with CAR-T cells or ICIs have not yet been reported, the underlying biology provides a compelling rationale. TGF-β1 is a major mediator of CAR-T dysfunction in solid tumors, promoting T-cell exhaustion, impaired trafficking and persistence, and regulatory T-cell differentiation, whereas HIF signaling further reinforces immune suppression within the hypoxic TME. Likewise, inhibition of the TGF-β1/HIF axis reduces PD-L1 expression, improves vascular normalization, and promotes immune-cell infiltration, thereby creating conditions favorable for immune checkpoint blockade. Together, these findings support a metabolic-first therapeutic strategy, in which SLM is used to reprogram the tumor microenvironment before or in combination with immunotherapy, providing a rational framework for overcoming resistance and improving durable clinical responses in ccRCC.

## 3. Characteristic of ccRCC with Sarcomatoid Differentiation

In the progression from conventional ccRCC to its sarcomatoid variant (srcRCC), the tumor undergoes a profound molecular “re-wiring.” This shift moves the cell from a state of metabolic storage and VHL-dependency to a state of mesenchymal aggression, genomic instability, and high glycolytic flux. SrcRCC is a highly aggressive variant of clear cell RCC characterized by sarcomatoid dedifferentiation through epithelial-to-mesenchymal transition. It is present in approximately 4–8% of all ccRCC cases and up to 15–25% of metastatic cases [17,18]. Any percentage of sarcomatoid component, even <1%, is sufficient for diagnosis and automatically assigns WHO/ISUP grade 4 with significantly worse prognosis (median overall survival 6–13 months in metastatic disease) [19,20,21,22,23,24]. Pathologically, srcRCC exhibits biphasic morphology: classic clear-cell areas admixed with spindle-cell sarcomatoid regions showing marked atypia, frequent necrosis, vimentin positivity, focal/low cytokeratin expression, and retained PAX8 expression [23]. Molecularly, srcRCC retains the core ccRCC driver events (VHL inactivation, chromosome 3p loss, PBRM1/SETD2/BAP1 mutations) but acquires additional alterations enriched in the sarcomatoid component, most notably TP53 mutations (42–60%), NF2 loss (19–30%), CDKN2A deletions, and ARID1A alterations [20,25,26,27]. These changes result in higher tumor mutational burden and an inflamed, PD-L1-high tumor microenvironment. Clinically, srcRCC displays high tumor mutational burden, PD-L1 expression, and neoantigen load, partly resulting from metabolic stress and oxidative DNA damage, explaining superior responses to immune checkpoint blockade (e.g., nivolumab + ipilimumab) compared with VEGF-TKIs, and shows poor response to VEGF-targeted therapies but significantly better outcomes with immune checkpoint inhibitors [28,29,30,31,32]. srcRCC is characterized by profound metabolic rewiring that fuels aggressive growth, immune evasion, and therapy resistance. Key metabolic hallmarks include Amplified Warburg effect (shift to aerobic glycolysis). Sarcomatoid areas exhibit marked preference for glycolysis even in oxygen-rich conditions, driven by persistent HIF-1α hyperactivity (from VHL inactivation plus additional EMT signals). This leads to strong upregulation of GLUT1, HK2, and LDHA. High expression of glutamine transporters and glutaminase supports TCA cycle, nucleotide synthesis, and redox balance, making sarcomatoid tumors highly glutamine-dependent. Despite loss of the classic “clear cell” lipid droplet appearance, sarcomatoid regions show increased de novo fatty acid synthesis and β-oxidation as alternative ATP sources under stress/hypoxia, as well as activation of the pentose phosphate pathway and one-carbon metabolism. These pathways supply NADPH for antioxidant defense and ribose-5-phosphate/methyl groups for rapid proliferation and DNA repair in highly mutagenic sarcomatoid cells [33,34,35].

Sarcomatoid differentiation in ccRCC is strongly associated with elevated expression of TGF-β1, HIF-1α, mutant p53, and PD-L1, alongside reduced expression of HIF-2α and VEGF compared with conventional ccRCC. HIF-1α is frequently expressed in the epithelial component of ccRCC with sarcomatoid transformation but is less frequently detected, or entirely absent, in the sarcomatoid component in approximately 50% of cases [36,37,38,39,40]. Similarly, HIF-2α is commonly retained in the epithelial regions, whereas sarcomatoid areas lack HIF-2α expression in a substantial proportion of tumors. This functional shift from HIF-2α to HIF-1α, compounded by hyperactive TGF-β1 signaling, p53 mutation, high PD-L1 expression, and reduced VEGF levels, constitutes a critical driver of the aggressive mesenchymal phenotype and the profoundly immunosuppressive tumor microenvironment characteristic of sarcomatoid ccRCC. These molecular features collectively contribute to resistance to both TKIs and ICIs. Consequently, durable therapeutic eradication of sarcomatoid ccRCC likely requires a pleiotropic strategy capable of simultaneously modulating these adverse prognostic pathways. In this context, pharmacologic inhibition of the TGF-β1/HIF axis by SLM, which is associated with downregulation of PD-L1, VEGF, and dismantling of the sarcomatoid system, offers a promising foundation for the development of novel therapeutic strategies. A comparative summary of the ccRCC and srcRCC profiles is shown in Table 1.

## 4. Standard of Care for Advanced Metastatic ccRCC

**Figure 1:**
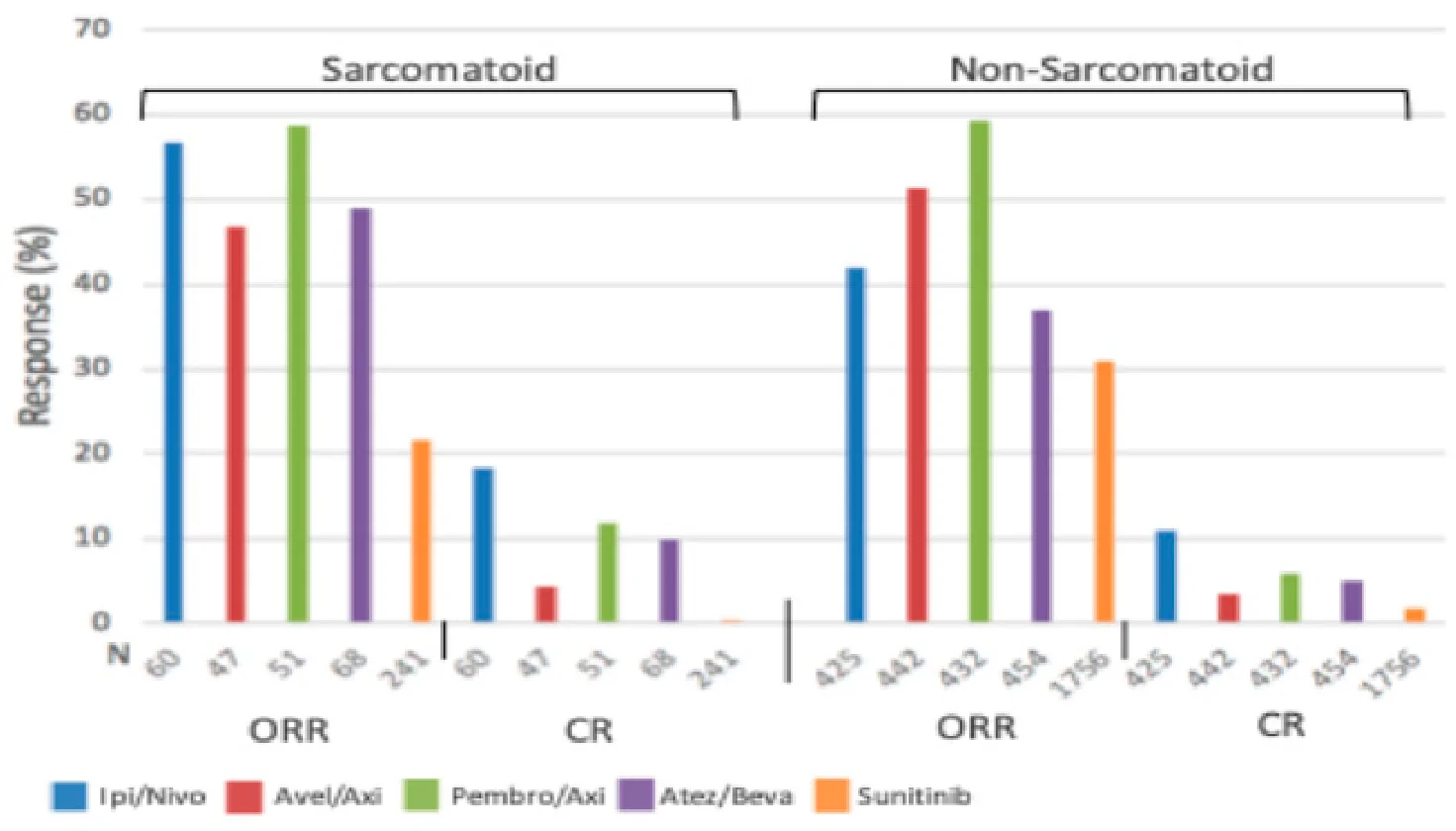
Randomized phase 111 clinical trials in previously untreated patients with advanced ccRCC with and without sarcomatoid differentiation. Ipilimumab/Nivolumab (Ipi/Nivo); Avelumab/Axitinib (Ave/Axi); Atezolizumab/Bevacizumab (Atex/Bev). N, number of patients in each response category [2].

The current landscape of treatment for ccRCC is defined by a shift away from traditional cytotoxic therapies toward targeted agents and immunotherapies, though significant barriers to long-term survival remain. For metastatic ccRCC, the five-year survival rate remains low, typically cited between 10% and 20%. While ICIs and TKIs have advanced treatment, over 50% of patients exhibit primary resistance to these therapies (Figure 1). In the 4 randomized phase 111 trials of newly diagnosed ccRCC patients, the reported objective response rate in non sarcomatoid and in sarcomatoid patients ranged from 38 to 58% and 45 to 58%, with complete response ranging from 0–1% and 0–18%, respectively. The preferred first line therapy, a combination of ICI with TKI, has demonstrated superior PFS, OS, and ORR compared to sunitinib across all risk groups. VEGF-TKI monotherapy is not recommended unless ICI is not recommended. In several clinical trials the combination of TKI with chemotherapy failed to demonstrate additional therapeutic benefit [1,2,3]. The transition from the failed cytotoxic era, exemplified by the inactivity of doxorubicin and gemcitabine, to a “metabolic-first” approach represents a paradigm shift in renal oncology. The innovation of metabolic priming lies in the sequence of therapy. By administering a metabolic deprogrammer like SLM first, we strip the ‘metabolic armor’ from the ccRCC cell, depleting lipid droplets and re-activating mitochondria, thereby converting a chemo-refractory tumor into a highly vulnerable target for standard cytotoxic or targeted agents.

Although pembrolizumab combined with axitinib has improved first-line outcomes in treatment-naïve ccRCC, durable responses remain limited due to persistent hypoxia signaling, metabolic adaptation, and TGF-β–driven immune suppression. Current immune checkpoint inhibitor and tyrosine kinase inhibitor strategies incompletely target upstream regulatory networks governing tumor plasticity and immune resistance. Pharmacologic SLM represents a mechanistically novel approach capable of simultaneously modulating TGF-β1/HIF signaling, restoring metabolic balance, and enhancing antitumor immune activity. Targeting these convergent master pathways may provide a rational strategy to potentiate and prolong responses to ICI–TKI therapy in ccRCC. The characteristic profile of ccRCC tumors that may provide the rationale for the development of novel combination is summarized in Table 2.

## 5. Liver and Lung Are Major Metastatic Organs in ccRCC

In ccRCC, the site of metastasis is one of the most significant predictors of how a patient will respond to immune checkpoint inhibitors (ICIs). The data in Table 3 represent a summary of the molecular profile of liver and lung tissue. There is a stark contrast between lung and liver metastases. Accumulating clinical evidence indicates that metastatic ccRCC patients with lung metastases exhibit more favorable responses and survival outcomes following immune checkpoint inhibitor-based therapy compared with patients harboring liver, bone, or brain metastases, highlighting the importance of organ-specific tumor immune microenvironments in determining therapeutic efficacy [41,42]. Lung metastases are typically “immunologically hot.” They tend to have higher densities of CD8+ T-cells and higher expression of PD-L1. In most ICI trials, the presence of lung-only or lung-dominant metastasis is associated with a higher likelihood of achieving a complete response (CR). Patients with lung metastases have some of the longest survival rates in the metastatic setting, sometimes exceeding 5–10 years with modern therapy.

In contrast, liver metastases are considered a major negative predictive factor for ICI efficacy in ccRCC. The liver acts as an “immune sink.” It contains specialized macrophages (Kupffer cells) that can “siphon” or delete activated T-cells from the systemic circulation. Meta-analyses show that ccRCC patients with liver metastases have significantly worse Progression-Free Survival and overall survival when treated with ICIs compared to those without liver involvement. The Hazard Ratio for death in ccRCC patients with liver metastases is often reported as high as 2.47 (more than double the risk). Liver metastases often exhibit an “immune excluded” phenotype, where the TGF-1 signaling is extremely high (Table 3). By inhibiting TGF-ß1, SLM may “break” the liver’s immune-suppressive shield. This could potentially convert a “liver-cold” tumor into a “liver-hot” tumor, allowing ICIs to work in the very patients who currently have the worst prognosis.

Clear cell renal cell carcinoma demonstrates a strong predilection for visceral dissemination, with the lung representing the most common metastatic site and the liver among the major organs involved in advanced disease. Hepatotoxicity remains a major barrier to the clinical translation of CD70-targeted CAR-T cell therapy in ccRCC, arising from on-target activation of CAR-T cells within the hepatic immune niche rather than direct hepatocyte targeting. Pharmacologic-dose SLM uniquely converts NF-κB signaling in hepatocytes from a pro-inflammatory driver of CAR-T-associated liver injury into a redox-buffered, cytoprotective stress-response program, preserving hepatic integrity without impairing CAR-T effector function. This may represent a first-in-class strategy to mitigate CAR-T hepatotoxicity by leveraging selenium-dependent NF-κB survival signaling rather than blunt cytokine suppression.

## 6. Therapeutic Potential of SLM in ccRCC with Liver and Lung Metastasis

The pharmacological distribution of selenium offers the potential for treating visceral metastases. The liver and lung represent two different metabolic environments, but both are uniquely sensitized by SLM. The liver is the body’s primary hub for selenium sequestration and processing. After oral administration of SLM, the liver receives the highest initial concentration via the portal vein. It is the site where SLM is converted into Selenoprotein. and other key metabolites. The lung has a lower absolute concentration of selenium compared to the liver, but it benefits from high blood flow and different functional kinetics. Lung tissue selenium levels are generally 30–50% of liver levels [43,44,45].

The differential levels of selenium in liver vs. lung metastasis justify why SLM is an ideal partner for pembrolizumab/axitinib in relapsed patients with visceral disease. By exploiting the liver’s role as a selenium processing hub, our strategy achieves the necessary pharmacological concentrations to degrade TGF-ß1/DNMT1 and re-awaken mitochondrial oxidative metabolism (SDH/FH) in liver metastases, an organ traditionally resistant to all forms of standard therapy. Concurrently, moderate lung distribution ensures a suppression of the HIF/VEGF.

Liver and lung metastasis would benefit from the oral administration of pharmacologic dose of SLM. Increase selenium tissue concentrations in liver will enhance TGF-ß1/HIF1α/DNMT1 inhibition resulting in the activation of SDH/FH, destabilization of HIF and reduction in the expression of PD-L1/VEGF. An increase in selenium concentrations in lung tumors will inhibit TGF-ß1 HIF2α, decrease the expression levels of PD-L1/VEGF, and increased the efficacy of immune/TKI in both liver and lung tumors. Following oral administration, seleno-L-methionine preferentially accumulates in the liver, the primary site of selenium metabolism, achieving high local pharmacologic concentrations capable of reversing TGF-β-driven immune exclusion via DNMT1 degradation and SDH/FH reactivation, while lower but kinetically favorable selenium levels in the lung suppress HIF-mediated VEGF and PD-L1 transcription, maintaining a permissive immune microenvironment.

## 7. Metabolic, Pathologic, and Molecular Characterization of Clear Cell Renal Cell Carcinoma

ccRCC represents approximately 80% of all renal malignancies and remains a leading cause of cancer-related death. Although significant advances have been achieved with targeted therapy, innate and acquired resistance and target toxicity continue to represent major clinical challenges. Better understanding of the multiplicity of factors altered in ccRCC tumors could provide the basis for the development of mechanism-based combination. Although the mechanisms of resistance of ccRCC to standard of care are multifactorial and complex, the key targets altered in human ccRCC biopsies and in clinically relevant cell lines are summarized in Table 1 and Table 2. The data in Table 1 and Table 2 represents a summary of the metabolic, pathologic, and molecular characteristics of primary and metastatic ccRCC and the associated mechanisms and translational potential.

### 7.1. Metabolic Reprogramming

**Figure 2:**
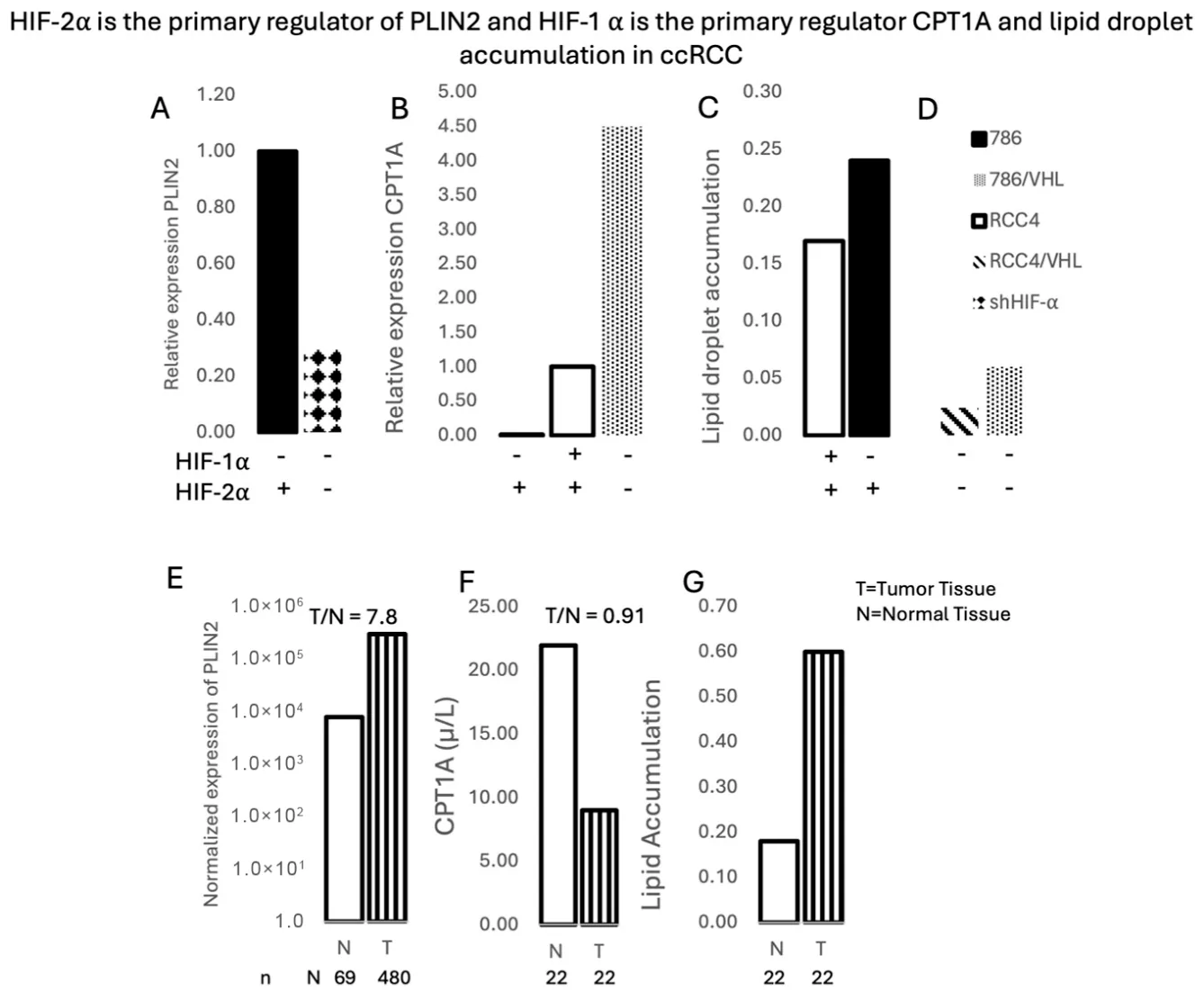
Correlation of HIF2A expression with the expression of PLIN2, CPT1A, and lipid droplet accumulation in 786-0, Figure (**A**–**D**), and PLIN2 and CPT1A expression in normal and tumor kidney tissue, Figure (**E**–**G**). The data in each panel were summarized from data indicated by each reference [46,50].

In ccRCC, primarily HIF-2α and secondarily TGFB1 regulate distinct metabolic genes, contributing to the disease’s metabolic reprogramming. HIF-2α is a transcription factor that responds to low oxygen levels (hypoxia), constitutively active in ccRCC due to VHL gene inactivation, and regulates genes involved in angiogenesis, metabolism, and survival, including those promoting lipid storage. PLIN2 i regulates lipid storage and prevents lipolysis. In ccRCC, PLIN2 is upregulated by HIF-2α, promoting lipid droplet accumulation, maintaining endoplasmic reticulum homeostasis, and supporting tumor cell viability by buffering against ER stress. CPT1A is the rate-limiting enzyme on the outer mitochondrial membrane that facilitates the transport of long-chain fatty acids into mitochondria for β-oxidation (fatty acid breakdown to produce energy). In ccRCC, HIF-2α (and HIF-1α) represses CPT1A expression, reducing fatty acid oxidation and diverting fatty acids toward lipid droplet storage. Specifically: HIF-1α directly binds to the CPT1A promoter and represses its transcription. Treatment with DMOG under normoxic condition stabilizes HIF1α, increases its binding to the CPT1A promoter, and decreases CPT1A mRNA levels, confirming that HIF activation is sufficient for this suppression without low oxygen. This repression reduces fatty acid transport into mitochondria, promotes lipid accumulation, and shifts cellular metabolism away from β-oxidation, which has been observed in ccRCC [46,47]. In patients with ccRCC, CPT1A expression was significantly lower in tumor tissue than in normal kidney tissue (relative expression approximately 48 vs. 65, *p* < 0.0001), and high CPT1A expression was associated with significantly longer overall survival than low CPT1A expression [48]. A lower level of CPT1A was reported to promote lipid accumulation in ccRCC [49]. HIF-2α regulates PLIN2, a lipid droplet-associated protein that stabilizes lipid droplets and contributes to the “clear cell” phenotype. Decreased CPT1A and activation of PLIN2 expression contribute to the characteristic lipid accumulation in clear cell RCC, supporting tumor growth by providing a reservoir of lipids for membrane synthesis and energy [47,50,51,52,53]. Collectively, the altered expressions of PLIN2 and CPT1A play a central role in immune evasion, resistance, and metabolic reprogramming, including lipid accumulation that contributes to the “clear cell” morphology. By stabilizing and activating HIFs, TGF-β1 directly or indirectly enhances PLIN2 upregulation, and mediates CPT1A repression, promoting lipid storage and metabolic shifts that fuel ccRCC growth [54,55,56]. HIF-2α and TGF-β1 stabilize lipid droplets via PLIN2 and CPT1A. In ccRCC, hypoxia-induced miR-210, upregulated via HIF-1α due to VHL loss, directly represses PLIN2 and CPT1A by binding their 3′UTRs, leading to reduced lipid droplet formation and impaired fatty acid β-oxidation, promoting lipid dysregulation and tumor progression [57]. This reprogramming interacts and suppresses ferroptosis (iron-dependent lipid peroxidation) in cells resistant to TKI. Overall, lipid accumulation acts as a metabolic barrier in the TME, driving CAR-T resistance by compromising T-cell viability and effector functions [58,59]. As demonstrated in Figure 2A–C, inhibition of HIF2 by shRNA and by transfection of 786-0 cells expressing high TGF and mutant VHL with functional VHL genes resulted in the down-expression of LPLIN2 and reduced lipid droplet accumulation. In ccRCC tumor biopsies, the under-expression of CPT1A is down-expressed and PLIN2 is upregulated in tumor tissue relative to normal kidney tissue—Figure 2D–G. Collectively, the data indicates that the overexpressed HIF2α functions as a primary regulator of the expression of CPT1A and PLIN2, which regulate lipid droplet accumulation in ccRCC tumor cells, and this process is reversed by the inhibition of HIF2 α. HIF-2α transcriptionally regulates lipid uptake and storage programs in ccRCC, driving expression of genes such as PLIN2, SCD1, and cholesterol transporters including SCARB1, thereby sustaining the lipid-laden phenotype characteristic of VHL-deficient tumors.

### 7.2. Pathologic Characterization

The hallmark “clear cell” appearance is due to lipid and glycogen accumulation in the cytoplasm. The TME is immunosuppressive, with high levels of TGF-β1, HIFs, and immunosuppressive cells, M2 macrophages, Tregs, and MDSCs [60,61,62,63,64,65,66,67]. This environment promotes resistance to immunotherapies and supports tumor progression. High expression of immune checkpoints, PD-L1 and CTLA-4, driven by HIF-1α/HIF-2α and TGF-β1, alongside lipid accumulation, creates an immunosuppressive TME in ccRCC that promotes immune evasion and resistance to CAR-T and checkpoint inhibitors. miR-210 amplifies this by sustaining hypoxia and glycolysis. Therapeutic strategies combining HIFs and TGF-β1 inhibitors by pharmacologic doses of SLM with checkpoint inhibitors, nivolumab and ipilimumab, hold promise in overcoming these barriers.

### 7.3. Molecular Characterization

The majority of advanced ccRCC tumors are characterized by the overexpression of TGF-β1 and by the stable expression of HIF-1α/2α due to VHL mutations. Both TGF-β1 and HIFs contribute to stable expression of PD-L1/VEGF/CTLA4 and miRNA-210. TGF-β1 differentially regulate the two enzymes involved in DNA methylation, activate DNMT and inhibit TET, leading to hypermethylation and tumor progression [12,68,69,70]. Activated DNMT1 regulates the expression of CD70 antigen target of CAR-T cells and inhibits PHD2, leading to stable expression of HIF, PD-L1, VEGF, and CTLA4, immune suppressive targets. Inhibition of DNMT promotes SDH and FH hypomethylation, restores SDH and FH activity, and mitigates TET and HIFs stabilization [71,72]. Restoring SDH activity via DNMT1 inhibition reduces succinate accumulation, reactivates α-KG-dependent enzymes (e.g., TET, PHD), and promotes HIF degradation, counteracting ccRCC progression. For FH, restored activity (if silenced epigenetically) would similarly reduce fumarate levels, alleviating inhibition of TET and PHD enzymes, though FH’s role in ccRCC is less prominent. The data in Table 2 represent a summary of the metabolic, pathologic and molecular profile of ccRCC.

### 7.4. Heterogeneity of Antigen Expression in ccRCC

Antigen expression in ccRCC is an important area of study for both diagnostic and immunotherapeutic purposes. Although several types of antigens are overexpressed in ccRCC, The Cluster of Differentiation 70 (CD70) is the primary antigen target for CAR-T cell therapy. CD70, a member of the TNF superfamily, is overexpressed in up to 90% of ccRCC tumors while showing limited expression in normal tissues, making it an ideal candidate for targeted immunotherapy to minimize off-tumor toxicity [72,73,74]. In ccRCC, HIF2α, NF-KB, TGF-β1, promoters of hypomethylation, namely activation of TET and inhibition of DNMT, pro-inflammations cytokines, INFg, TNFα, and PI3K/AKT-mTOR are reported as regulators of CD70 transcription and T-cell exhaustion [75,76,77]. Recent insights into the interconnected TGF-β1/HIF/miR-210 axis highlight a unifying mechanism that links metabolic reprogramming, epigenetic dysregulation, and immune suppression in ccRCC. Targeting this axis, particularly by restoring mitochondrial function, reducing oncometabolite accumulation, and destabilizing HIFs, offers a promising therapeutic framework to overcome entrenched resistance. Emerging approaches such as pharmacologic Seleno-L-methionine, which disrupts this axis at multiple nodes, underscore the feasibility and translational potential of metabolically focused combination strategies. Together, these advances suggest that reprogramming tumor metabolism may be key to unlocking durable immunotherapy responses in ccRCC.

## 8. Role of Oncogenic and Tumor Suppressor microRNAs in Metabolic Reprogramming of ccRCC

Clear cell renal cell carcinoma (ccRCC) is defined by profound metabolic reprogramming driven primarily by loss of VHL and constitutive activation of HIF-1α and HIF-2α. MicroRNAs (miRNAs) serve as critical post-transcriptional regulators of this metabolic phenotype, functioning either as oncogenic miRNAs that reinforce glycolytic, lipogenic, and hypoxia-adaptive programs, or as tumor suppressor miRNAs that normally restrain these pathways. Dysregulation of these miRNA networks directly contributes to mitochondrial suppression, lipid accumulation, and immune evasion in ccRCC. Among the most prominent oncogenic miRNAs in ccRCC is miR-210, a canonical HIF-induced “hypoxamiR.” miR-210 suppresses mitochondrial respiration by targeting iron–sulfur cluster assembly proteins (ISCU1/2) and key tricarboxylic acid (TCA) cycle components, thereby enforcing reliance on aerobic glycolysis and promoting lactate accumulation [76,77]. Elevated miR-210 expression is consistently associated with aggressive disease and poor clinical outcomes. Conversely, several tumor suppressor miRNAs that normally limit metabolic plasticity are downregulated in ccRCC. miR-34a, a p53-regulated miRNA, suppresses glycolysis and lactate production by targeting LDHA, MYC, and SIRT1, thereby favoring mitochondrial oxidative metabolism [78,79,80]. Loss of miR-34a enhances metabolic flexibility and supports tumor progression. Additional tumor suppressor miRNAs, including miR-199a and let-7 family members, further constrain HIF signaling, lipid uptake, and mitochondrial dysfunction, but are frequently silenced through epigenetic mechanisms. MiRNA-mediated metabolic control in ccRCC is tightly integrated with TGF-β1 and HIF signaling. Both pathways transcriptionally induce oncogenic miRNAs (e.g., miR-21, miR-210) while repressing tumor suppressor miRNAs (e.g., miR-34a, miR-199a), forming a feed-forward regulatory network that amplifies mitochondrial repression, lipid droplet accumulation, and resistance to immune-mediated killing.

Therapeutically, restoring tumor suppressor miRNAs or inhibiting oncogenic miRNAs represents a compelling strategy to reverse ccRCC metabolic reprogramming. miRNA mimics (e.g., miR-34a, miR-199a) or antagomiRs targeting miR-21 and miR-210 may synergize with HIF-2α inhibitors, tyrosine kinase inhibitors, immune checkpoint blockade, and metabolic modulators to overcome therapeutic resistance. Notably, our laboratory was the first to demonstrate in ccRCC cell lines and patient samples that pharmacologic doses of seleno-L-methionine (SLM) exert dual regulatory effects on miRNA expression, selectively upregulating tumor suppressor miRNAs (e.g., let-7b) while modulating oncogenic miRNAs such as miR-155 and miR-210. We propose that pharmacologic SLM functions as a first-in-class dual inhibitor of the TGF-β1/HIF axis, a central regulator of miRNA expression and epigenetic stability in ccRCC. By inhibiting this axis, SLM reduces DNMT activity and promotes DNMT1 degradation, enabling demethylation and re-expression of tumor suppressor miRNAs. Restored tumor suppressor miRNAs subsequently repress SCARB1 expression, limiting exogenous lipid uptake, while concurrent restoration of TET-mediated demethylation reactivates mitochondrial oxidative metabolism. Collectively, these effects reprogram the tumor cell from a lipid-dependent “sink” into a metabolically vulnerable target, enhancing susceptibility to CAR-T cell-based immunotherapies.

## 9. CAR-T Cell Therapy in ccRCC: Barriers and a Mechanistically Grounded Sensitization Strategy

While chimeric antigen receptor (CAR) T-cell therapy has demonstrated substantial clinical benefit in hematologic malignancies, its efficacy in ccRCC remains limited. Early-phase clinical studies indicate that CAR-T therapy is feasible in ccRCC and can induce antitumor activity in selected patients; however, responses are frequently not durable [72,73,74,75,76,77,78,79,80,81,82,83]. These outcomes underscore persistent biological barriers unique to ccRCC, including intratumoral antigen heterogeneity, restricted T-cell infiltration, and progressive CAR-T dysfunction within an immunosuppressive and metabolically adverse tumor microenvironment (TME). Accumulating evidence indicates that TGF-β1 and HIF-1α/2α signaling represent central regulators of immune resistance in ccRCC. These pathways promote immune exclusion, enforce metabolic constraints on effector lymphocytes, and sustain the expression of inhibitory immune checkpoints, including CTLA-4. Consequently, CAR-T cells entering the ccRCC TME are exposed to persistent, hypoxia-mediated suppression, resulting in impaired effector function, limited persistence, and premature exhaustion. These dominant resistance mechanisms are not adequately addressed by current CAR design strategies. Various approaches have been pursued to improve CAR-T efficacy in ccRCC, including multi-antigen targeting (e.g., CD70, CAIX) to mitigate antigen escape, and checkpoint-modified CAR constructs designed to resist PD-L1-mediated inhibition [84,85,86,87,88,89]. In parallel, low-dose, metronomic chemotherapy has been shown to partially normalize tumor vasculature and modulate immune suppression, whereas conventional high-dose regimens may exacerbate hypoxia and stromal activation. While these approaches address discrete components of resistance, none directly target the integrated metabolic and checkpoint-driven constraints imposed by the ccRCC TME. Emerging preclinical evidence that pharmacologic doses of SLM selectively inhibit TGF-β1 and HIF signaling advances a mechanistically integrated strategy to enhance CAR-T function in ccRCC through targeted, non-genetic interventions. We propose that ex vivo exposure of T cells to effective doses of SLM during manufacturing serves as a metabolic conditioning strategy to attenuate TGF-β/HIF signaling prior to tumor engagement. This approach is expected to improve mitochondrial fitness and resistance to exhaustion, thereby enhancing CAR-T effector capacity. Concomitant in vivo SLM administration is intended to sustain and expand CAR-T persistence by reducing tumor-derived TGF-β1/HIFs and regulating VEGF and CTLA-4, thereby preserving effector function within the hypoxic ccRCC TME. We anticipate that pre-treatment with Methylseleninic acid (MSA) will further improve the persistence and efficacy of CAR-T cells expressing dual CD70/Fn14 antigens, as this ex vivo priming establishes the cellular fitness required for subsequent in vivo treatment with SLM. SLM is also proposed to function as a modulator capable of restoring tumor antigen expression while suppressing checkpoint pathways. Through the inhibition of DNA methyltransferase activity and indirect suppression of TGF-β-associated epigenetic silencing, SLM may upregulate the CD70 promoter in tumors with low or heterogeneous antigen expression. In parallel, reduced expression of PD-L1 and CTLA-4 may facilitate the development of memory-like CAR-T phenotypes associated with improved durability. Finally, integrating SLM with low-dose chemotherapy, using agents such as cisplatin and decitabine known to upregulate antigen expression and partially suppress TGF-β/HIF signaling, is proposed to further normalize the ccRCC TME. Overall, these approaches establish a clinically feasible, non-genetic sensitization framework for CAR-T therapy that directly targets the TGF-β/HIF resistance axis in ccRCC, with the potential to substantially improve CAR-T persistence and therapeutic durability in solid tumors.

## 10. Major Obstacles and Challenges to CAR-T Cell Therapy in Solid Tumors

While chimeric antigen receptor (CAR) T-cell therapy has produced unprecedented clinical responses in hematologic malignancies, particularly B-cell leukemias and lymphomas [74,83,84,85,86], translating this success to solid tumors has proven considerably more challenging. Solid tumors present complex biological, immunological, metabolic, and physical barriers that collectively impair CAR-T cell trafficking, infiltration, persistence, and cytotoxic function [87,88,89,90,91,92,93,94,95,96,97,98]. Comprehensive reviews have identified antigen heterogeneity, poor tumor infiltration, T-cell exhaustion, metabolic dysfunction, and immunosuppressive tumor microenvironments (TME) as the principal barriers limiting CAR-T efficacy in this setting. A primary limitation is the lack of highly tumor-specific target antigens. Unlike hematologic malignancies, most solid tumor-associated antigens are also expressed at varying levels in normal tissues, creating a risk of on-target/off-tumor toxicity. In clear cell renal cell carcinoma (ccRCC), CD70 and fibroblast growth factor-inducible 14 (Fn14; TNFRSF12A) are considered promising targets due to their high prevalence and relatively restricted expression in normal tissues. Although recent studies confirm that CD70 is consistently expressed in both primary and metastatic ccRCC lesions, intratumoral and interpatient heterogeneity in antigen expression remains a significant challenge that may promote antigen escape and limit the durability of CAR-T responses. Solid tumors are often anatomically restricted and difficult for CAR-T cells to penetrate. Abnormal tumor vasculature, elevated interstitial fluid pressure, extracellular matrix deposition, hypoxia, and dense stromal barriers collectively impede CAR-T trafficking and infiltration, thereby reducing effective contact with tumor cells and limiting intratumoral persistence. These barriers are particularly prominent in highly desmoplastic and immunosuppressive tumors, including pancreatic cancer, glioblastoma, and advanced ccRCC. Consequently, recent translational and clinical studies continue to identify inadequate trafficking and persistence as major determinants of treatment failure. The TME represents a formidable obstacle to CAR-T efficacy, containing multiple immunosuppressive factors—such as TGF-β1, HIF-1α/HIF-2α, PD-L1, CTLA-4, regulatory T cells (Tregs), myeloid-derived suppressor cells (MDSCs), and tumor-associated macrophages (TAMs). Collectively, these factors promote T-cell exhaustion, suppress proliferation and cytokine production, impair metabolic fitness, reduce persistence, and ultimately diminish antitumor activity. Emerging evidence indicates that metabolic stress and chronic antigen stimulation work synergistically to drive CAR-T dysfunction and exhaustion.

In summary, CAR-T therapy in solid tumors faces multiple interconnected barriers, including antigen heterogeneity, inadequate trafficking and infiltration, immunosuppressive tumor microenvironments, T-cell exhaustion, and metabolic dysfunction. In ccRCC, therapeutic strategies that simultaneously target the TGF-β/HIF immunometabolic axis and tumor-associated antigens such as CD70 and Fn14 may provide a promising approach to enhance CAR-T cell persistence, tumor infiltration, and durable antitumor responses. This combinatorial approach may ultimately help overcome several of the fundamental limitations that have thus far restricted the success of CAR-T therapy in solid tumors.

## 11. Role of SLM in Normalizing Metabolic Distinction Between ccRCC and srcRCC

The srcRCC variant represents the most aggressive, “metabolically extreme” end of the ccRCC spectrum. It is characterized by a complete transition to a mesenchymal, glycolytic state with massive overexpression of Fn14 and PD-L1. The role of SLM in leveraging CAR-T efficacy differs significantly between these two phenotypes based on their baseline metabolic and antigenic density. 1. srcRCC tumor has undergone a full epithelial-to-mesenchymal transition (EMT). In srcRCC high Fn14 levels drive extreme TKI bypass, while massive PD-L1 and CD70 expression create an immediate “exhaustion wall” for CAR-T cells. SLM acts as a structural and antigenic de-bulker by suppressing the TGF-/Fn14 axis and physically “softens” the tumor, reducing interstitial pressure and allowing CAR-T cells to penetrate the sarcomatoid nests. It prevents “terminal exhaustion” by lowering the overwhelming PD-L1/CD70 signal to a level the T-cells can survive. 2. Non-Sarcomatoid ccRCC (Clear Cell): the tumor is defined by massive lipid and glycogen storage (the “Clear Cell” look). The “Lipid Sink” (PLIN2/SCARB1) and high succinate levels paralyze CAR-T metabolism. CD70 is high but lacks the mesenchymal Fn14-driven invasiveness of srcRCC. In non-sarcomatoid ccRCC, SLM acts as a metabolic resuscitator by reactivating SDH/FH and forces the tumor to consume its lipid stores, removing the metabolic toxins that inhibit CAR-T mitochondrial function. While ccRCC and srcRCC are traditionally viewed as metabolically and therapeutically distinct entities, this dichotomy is largely sustained by divergent activation of the TGF-β and HIF signaling networks. We posit that pharmacologic doses of seleno-L-methionine (SLM), through pleiotropic and coordinated suppression of both TGF-β and HIF pathways, can collapse these metabolic distinctions. By overriding lineage-specific metabolic wiring and enforcing a shared, therapy-permissive state, SLM represents a first-in-class metabolic reprogramming strategy capable of converting srcRCC from an intrinsically refractory phenotype into one that is responsive to targeted agents developed for ccRCC.

## 12. Discussion

Cancer therapy has evolved beyond conventional modalities, such as surgery, chemotherapy, and radiation, toward precision approaches including targeted therapy, ICIs, TKI and CAR-T cell therapy, which selectively eliminate tumor cells while minimizing damage to normal tissues. Although CAR-T cell therapy has achieved remarkable success in hematologic malignancies and has shown encouraging activity in brain tumors and ccRCC [74,83,85,86], and ICIs have transformed the treatment of several solid tumors, durable responses remain limited because most solid tumors develop adaptive mechanisms of immune escape and therapeutic resistance.

A central driver of this resistance is the immunosuppressive tumor microenvironment (TME), in which persistent TGF-β1 signaling and HIF stabilization cooperatively promote immune evasion, metabolic adaptation, angiogenesis, and resistance to therapy through upregulation of PD-L1, CTLA-4, VEGF, and other downstream effectors. This review highlights the therapeutic potential of pharmacologic-dose seleno-L-methionine (SLM) as a pleiotropic immunometabolic checkpoint modulator that disrupts the TGF-β1/HIF axis and restores antitumor immunity.

The antitumor activity of SLM is mediated through multiple complementary mechanisms. Pharmacologic-dose SLM suppresses TGF-β1 signaling and indirectly inhibits DNA methyltransferase (DNMT) activity through HIF downregulation, while restoring prolyl hydroxylase domain protein 2 (PHD2) activity, thereby promoting HIF hydroxylation and proteasomal degradation. Attenuation of HIF signaling consequently decreases the expression of PD-L1, CTLA-4, and VEGF, suppressing immune evasion, angiogenesis, and tumor progression. In ccRCC, HIF-2α transcriptionally upregulates CD70, an emerging CAR-T cell target, whereas DNMT-mediated promoter hypermethylation can silence CD70 expression and reduce tumor immunogenicity. By degrading DNMT1 and promoting DNA demethylation, SLM may restore CD70 expression, enhance tumor recognition by CAR-T cells, and further alleviate PD-L1- and CTLA-4-mediated immunosuppression.

At clinically achievable pharmacologic concentrations, SLM exerts five complementary antitumor activities: (1) epigenetic reprogramming through coordinated inhibition of DNMTs and activation of TET dioxygenases; (2) suppression of immunosuppressive mediators, including PD-L1, VEGF, and CTLA-4; (3) normalization of the tumor vasculature, improving immune cell infiltration and drug delivery; (4) protection of normal tissues from chemotherapy-induced toxicity; and (5) enhancement of the efficacy of cytotoxic chemotherapy, targeted therapies, ICIs, and CAR-T cell therapy.

Importantly, the therapeutic activity of SLM may not be restricted to tumors with high TGF-β1/HIF activity. In approximately 15% of ccRCC tumors with relatively low TGF-β1 and/or HIF activity, SLM may retain activity through TGF-β1/HIF-independent metabolic and epigenetic mechanisms, including modulation of redox homeostasis, ferroptosis susceptibility, DNMT1 expression, and DNA methylationImportantly, the therapeutic activity of SLM is not restricted to tumors with high TGF-β1/HIF expression [99,100,101,102,103].

**Figure 3:**
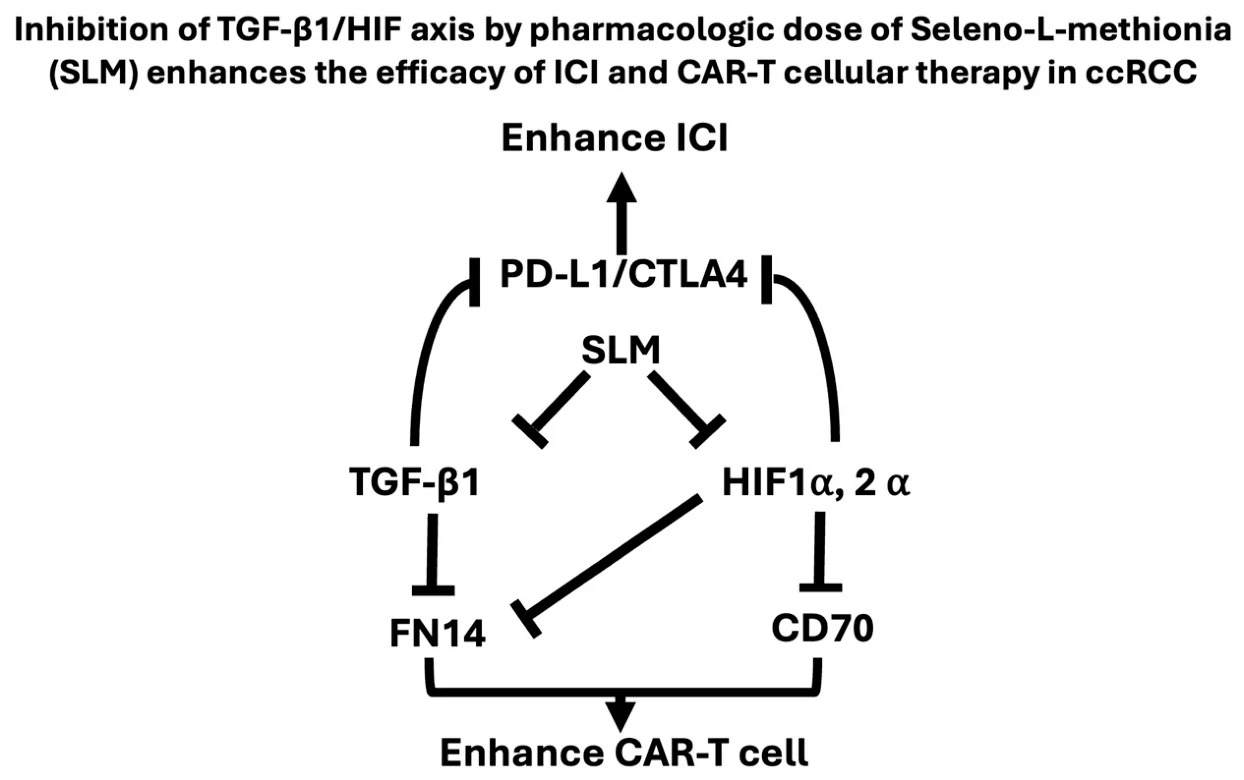
Schematic representation for the therapeutic potential of inhibiting transforming growth factor 1 (TGF- β1) hypoxia-inducible factor 1a and 2a by pharmacologic Seleno-L-methionine (SLM); immune checkpoint inhibitors (ICI); programmed death ligand 1 (PD-L1); cytotoxic T-lymphocyte antigen 4 (CTLA4); Fibroblast Growth Factor-Inducible 14/TWEAK Receptor (Fn14); A member of the tumor necrosis factor superfamily. It is the ligand for CD27 (CD70). Arrow activation; slash inhibition.

Collectively, the available evidence supports a dual mechanism of action for SLM. In TGF-β1/HIF-driven tumors, SLM disrupts the central immunometabolic resistance axis by promoting degradation of TGF-β1/HIF signaling and its downstream effectors. In tumors with lower TGF-β1/HIF activity, SLM maintains antitumor efficacy through metabolic and epigenetic reprogramming, including restoration of mitochondrial function, activation of ferroptosis, and reversal of aberrant DNA methylation. By dismantling lipid droplet-mediated metabolic buffering and reversing immunometabolic plasticity, SLM has the potential to sensitize ccRCC to chemotherapy, targeted agents, ICIs, and CAR-T cell therapy, providing a strong mechanistic rationale for its clinical evaluation as a broadly applicable adjunct to combination cancer immunotherapy (Figure 3).

## 13. Therapeutic and Translational Potential

The therapeutic and translational potential of targeting the TGF-β1/HIF axis with pharmacologic-dose SLM is supported by its ability to reverse the immunometabolic reprogramming that drives therapeutic resistance in ccRCC. Mechanistically, SLM inhibits TGF-β1, HIF-1α/HIF-2α, and miR-210, resulting in downregulation of VEGF and PLIN2, activation of CPT1A, restoration of mitochondrial oxidative metabolism, and a metabolic shift from glycolysis toward fatty acid oxidation. These coordinated effects reduce tumor hypoxia and immunosuppression while enhancing T-cell infiltration, effector function, and persistence.

In multiple preclinical models, pharmacologic-dose SLM administered using an optimized dose and schedule in combination with chemotherapy or targeted therapies demonstrated minimal off-target toxicity and significant synergistic antitumor activity. These findings provided the scientific rationale for the clinical evaluation of SLM combined with axitinib in previously treated patients with advanced ccRCC, where encouraging clinical activity was observed [104].

By functioning as a first-in-class immunometabolic modulator, SLM remodels both tumor cell metabolism and the tumor microenvironment, providing a strong mechanistic rationale for combination strategies with immune checkpoint inhibitors, antiangiogenic agents, and adoptive cellular therapies. In particular, by improving tumor perfusion, reducing lipid-mediated immune dysfunction, normalizing the tumor microenvironment, and enhancing T-cell fitness, SLM has the potential to improve CAR-T cell trafficking, persistence, and antitumor efficacy in ccRCC and other solid tumors. Figure 3 schematically illustrates the therapeutic and translational potential of dose- and schedule-dependent inhibition of the TGF-β1/HIF axis by pharmacologic SLM, highlighting its capacity to overcome immunometabolic resistance and enhance multimodal cancer therapy.

## 14. Key Innovation and Future Perspectives

The central innovation of this review is the proposal that pharmacologic dose- and schedule-dependent administration of SLM can function as a systems-level immunometabolic modulator rather than as a conventional selenium supplement or single-target inhibitor. Our preclinical and clinical studies demonstrated that optimal pharmacologic dosing of SLM simultaneously suppresses TGF-β1 and promotes degradation of HIF-1α/HIF-2α, thereby disrupting a master regulatory network that coordinates immune evasion, metabolic reprogramming, angiogenesis, and therapeutic resistance in ccRCC. Unlike current targeted therapies that inhibit individual downstream pathways and are frequently circumvented by compensatory signaling, SLM targets the upstream TGF-β1/HIF axis, leading to pleiotropic biological effects. These include normalization of tumor vasculature, improved intratumoral drug delivery, reversal of immunosuppression, restoration of mitochondrial metabolism, reduction in lipid-droplet accumulation, epigenetic reprogramming, and enhanced susceptibility of tumor cells to immune-mediated killing. Importantly, these effects are strictly dose- and schedule-dependent, highlighting the importance of pharmacologic optimization rather than nutritional selenium supplementation.

This systems-level reprogramming also provides a unique translational platform for immunotherapy. In vivo SLM remodels the tumor microenvironment by reducing TGF-β1/HIF-driven barriers to immune infiltration, whereas ex vivo methylseleninic acid, the active metabolite of SLM, metabolically reprograms CAR-T cells to improve persistence, trafficking, and cytotoxicity. Together, these complementary approaches target both the tumor and the immune effector cell, providing a mechanistic rationale for combining SLM with immune checkpoint inhibitors, antiangiogenic agents, and dual-target CAR-T therapies.

Rather than functioning as another targeted agent, SLM represents a first-in-class pharmacologic reprogramming strategy designed to dismantle the TGF-β1/HIF-driven immunometabolic network that underlies resistance in ccRCC. The encouraging preclinical evidence and early clinical activity support further mechanistic studies and prospective randomized clinical trials to validate SLM as a platform therapy capable of enhancing multiple treatment modalities and improving long-term outcomes in patients with advanced ccRCC.

## 15. Take Home Message

We were the first to demonstrate, in both in vitro and in vivo xenograft models and subsequently in patients with advanced ccRCC, that the simultaneous inhibition of TGF-β1 and enhancement of HIF-1α/HIF-2α degradation by selenium is highly dose- and schedule-dependent [105,106,107,108,109,110]. This pharmacodynamic response was associated with downregulation of PD-L1 and VEGF [108,110], normalization and stabilization of the tumor vasculature, and selective enhancement of anticancer drug delivery to tumors while sparing normal tissues [111,112]. Collectively, these pleiotropic effects of SLM translated into durable therapeutic responses, including long-term survival in xenograft models treated with chemotherapy [113,114] and encouraging clinical outcomes in patients with advanced ccRCC treated with SLM plus axitinib [104]. Importantly, ex vivo treatment of Fn14 CAR-T cells with methylseleninic acid (MSA), the active metabolite of SLM, inhibited the TGF-β1/HIF signaling axis and significantly enhanced CAR-T cell persistence, activation, and cytotoxicity [115,116]. These findings support a unified translational strategy in which in vivo SLM remodels the immunosuppressive tumor microenvironment, while ex vivo MSA metabolically and functionally reprograms CAR-T cells, thereby targeting both the tumor and the immune effector cell. Taken together, the mechanistic and therapeutic evidence from preclinical studies, combined with the encouraging clinical activity of SLM plus axitinib in patients with advanced ccRCC, provides a strong scientific rationale for further investigation of SLM as a pleiotropic immunometabolic modulator. Prospective randomized clinical trials are now warranted to validate its ability to enhance the efficacy of immune checkpoint inhibitors, tyrosine kinase inhibitors, and CAR-T cell therapy in ccRCC.

## Figures and Tables

**Table 1:** Summary of the ccRCC molecular switch.

Parameter	Conventional ccRCC	Sarcomatoid RCC (sarRCC)
Dominant HIF	HIF2α	HIF1α
Lipid status	High PLIN2 (lipid droplets)	Low PLIN2 (Spindled/fibrotic)
TGF-1 levels	Moderate	High/extreme
Metabolism	Balanced glycolysis/storage	High glycolytic flux
Therapeutic target	HIF2α/VEGF	TGF-1/HIF1α/IO

**Table 2:** Characterization of ccRCC.

Aspect	Key Features & Markers	Mechanisms/Pathways	Clinical Implications
Metabolic	- Lipid accumulation (PLIN2 ↑, CPT1A ↓) - Glycolysis ↑ - Succinate, fumarate ↑	- VHL loss → HIF-1α/2α stabilization - HIFs repress CPT1A, activate PLIN2 - SDH/FH deficiency	- “Clear cell” morphology - Immune evasion - Therapy resistance
Pathologic	- Clear cytoplasm - Lipid/glycogen-rich cells - Immunosuppressive TME	- TGF-β1/HIF axis promotes EMT, fibrosis, immune suppression - High PD-L1, CTLA-4 expression	- Poor response to immunotherapy - Tumor progression
Molecular	- VHL mutation (most cases) - TGF-β1/HIF-1α/2α ↑ - miR-210 ↑ - DNMT1 ↑, TET ↓	- TGF-β1/HIFs drive metabolic reprogramming - miR-210 suppresses mitochondrial function - Epigenetic silencing of SDH/FH	- Predictive biomarkers - Targetable vulnerabilities

Legend: ↑ = upregulated/increased, ↓ = downregulated/decreased, TME = Tumor microenvironment, EMT = Epithelial–mesenchymal transition, SDH = Succinate dehydrogenase. FH = Fumarate hydratase. PLIN2 = Perilipin 2, CPT1A = Carnitine palmitoyl transferase 1A. PD-L1, CTLA-4 = Immune checkpoint proteins, DNMT1 = DNA methyltransferase 1, TET = Ten-eleven translocation enzymes, miR-210 = microRNA.

**Table 3:** Molecular characterization of ccRCC liver and lung metastasis.

	Liver Metastasis	Lung Metastasis
Selenium concentrations	High	50% lower
TGF-ß1	High	Low
HIF1α	Low	High
HIF2α	Low	High
PD-L1	High	High
VEGF	High	High

## Data Availability

No new data were created or analyzed in this study. Data sharing is not applicable to this article.
